# A Combination of Species Identification and STR Profiling Identifies Cross-contaminated Cells from 482 Human Tumor Cell Lines

**DOI:** 10.1038/s41598-017-09660-w

**Published:** 2017-08-29

**Authors:** Xiaocui Bian, Zhenli Yang, Hailiang Feng, Hao Sun, Yuqin Liu

**Affiliations:** Department of Pathology, Cell Resource Center, Institute of Basic Medical Sciences, Chinese Academy of Medical Sciences & School of Basic Medicine, Peking Union Medical College, Beijing, 100005 P. R. China

## Abstract

Human tumor cell lines are extremely important tools for cancer research, but a significant percentage is cross-contaminated with other cells. Short tandem repeat (STR) profiling is the prevailing standard for authenticating cell lines that originate from human tissues. Based on the analysis of 482 different human tumor cell lines used in China by STR, up to 96 cell lines were misidentified. More importantly, the study has found that STR profiling alone is insufficient to exclude inter-species cross-contamination of human cell lines. Among the 386 cell lines which had a correct STR profile, 3 of them were inter-species cross-contaminated. Careful microscopic examination may be helpful in some cases to detect changes in morphology but additional testing is needed. Additionally, species verification by PCR could easily identify the contaminants, even with a low percentage of contaminating cells. Combining STR profiling with species identification by PCR, more than 20.5% (99/482) of tumor cell lines were revealed as having been incorrectly identified, including intra-species (14.5%), inter-species (4.4%) cross-contamination and contaminating cell lines (1.7%). Therefore, quality control of cell lines is a systemic issue. Each cell line should undergo a full QA (Quality Assurance) assessment before it is used for research.

## Introduction

*In vitro*-cultured human tumor cell lines are extremely important tools for unraveling the mechanisms of tumor development, progression and metastasis, and discovering new therapies. The use of authenticated tumor cell lines holds important translational value for their model character and their reproducibility in the clinical area. However, most of currently used tumor cells are continuous cell lines, and a foreign cell line or microorganism is easily introduced without the handler’s knowledge during continuous passage culture. So each cell line should undergo a full QA assessment before it is used for research. The quality analysis of cell lines typically includes microorganism detection and species verification; also, to double-check their identity, STR profiling of human-derived cell lines should be combined with routine examination of their morphological and specific properties^1–3^. Over the past 10 years, we have been performing all QAs for quality control of the cell lines in our center (China Infrastructure of Cell Line Resource, CICR). We also provide QA services for the cell lines of other laboratories. Most customers only chose STR profiling for human cell lines, considering it to be the gold standard^2–7^. However, STR profiling is occasionally insufficient. In this paper, we share our experience and our view.

## Results

### Overview of the tested cell lines

**Figure 1:**
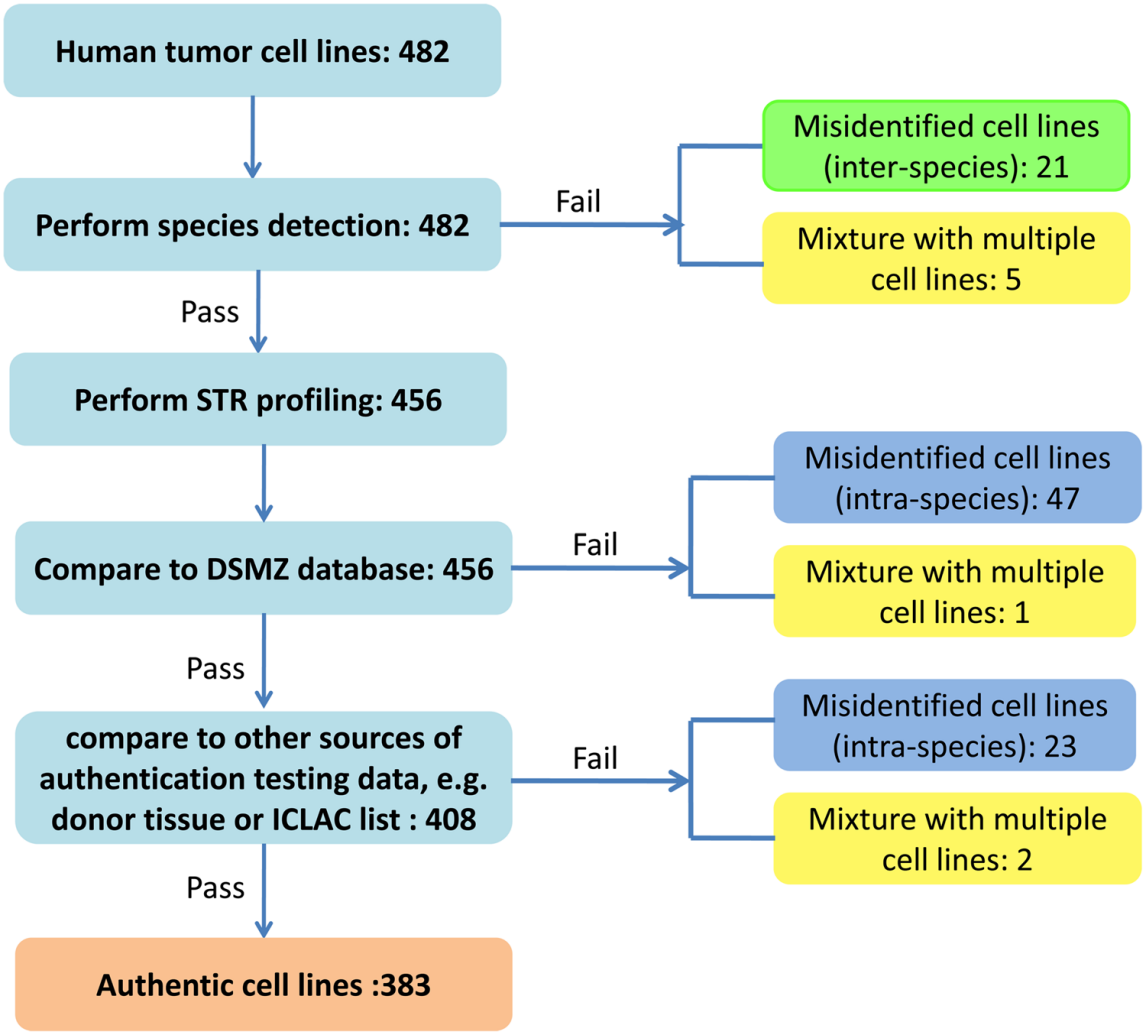
Workflow for authentication of human tumor cell lines.

**Figure 2:**
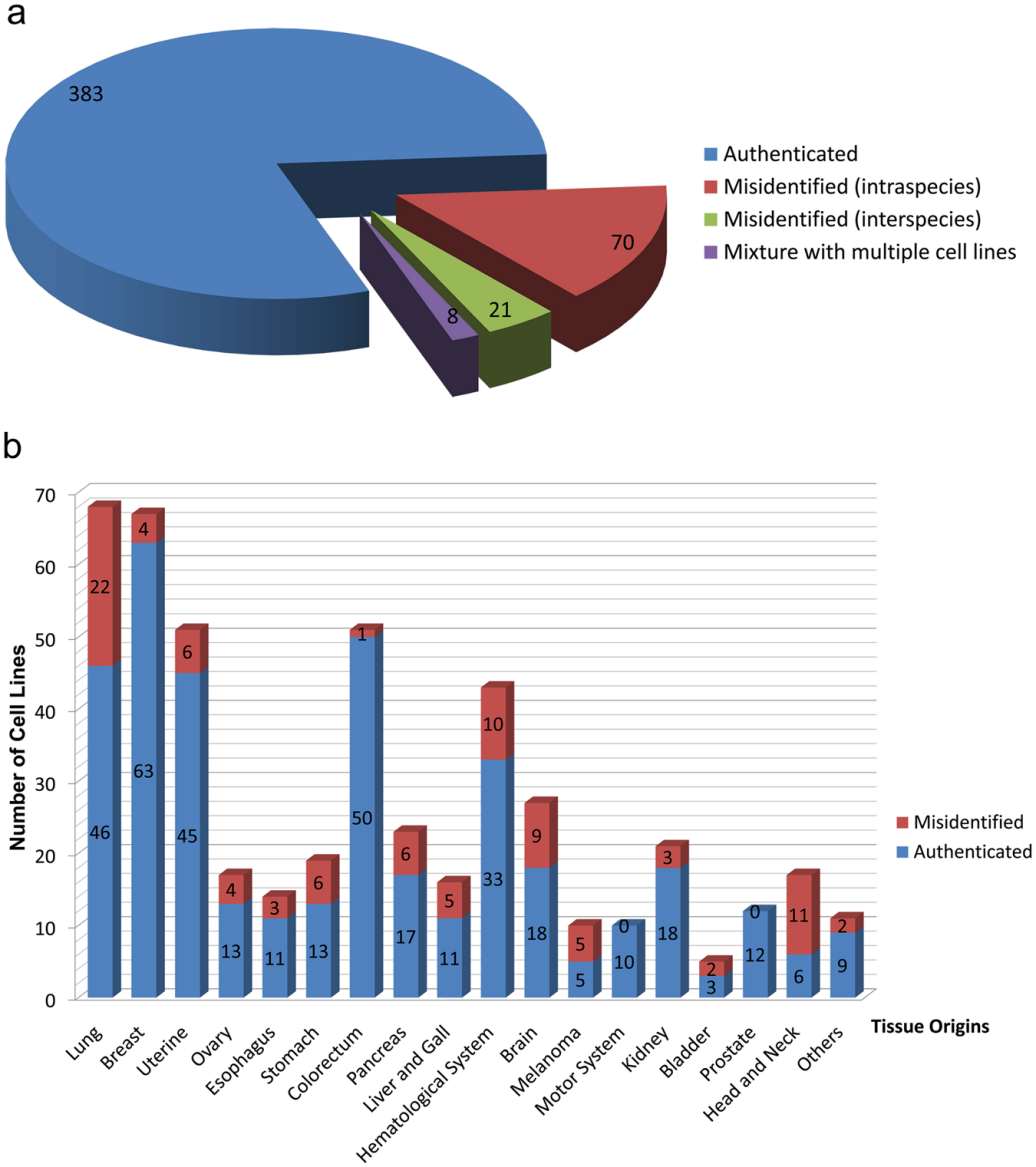
Overview of the 482 cell lines tested. (**a**) The pie graphs show cases of authenticated, intra- and inter-species cross-contamination of human tumor cell lines. (**b**) All tumor cells were divided into 18 categories according to their tissue origins and cases of authenticated and misidentified cell lines of each category are showed in the column chart. Misidentified cell lines include intra-species and inter-species cell lines.

We obtained data for the species verification and STR profiles of 482 different human tumor cell lines which derived from patients with a variety of tumors (Fig. 1). Among the 482 cell lines, as illustrated in Figs. 1 and 2a, 79.5% (383) (see Supplementary Table S1) were authenticated cells, more than 14.5% (71) were the correct species with the wrong STR, 4.4% (21) were the wrong species and 1.7% (8) were mixtures of multiple cell lines at present. These 4 classes of cell lines will be described in detail below. All 482 tumor cells were classified into 18 categories according to their tissue origins: breast cancers (68 cell lines), lung cancers (67 cell lines), uterine tumors (51, including 30 cervical carcinoma cells, 16 endometrial adenocarcinoma cells and 5 choriocarcinoma cells), ovarian carcinomas (17), esophageal carcinomas (14), gastric carcinomas (19), colorectal carcinomas (51), pancreatic carcinomas (23), liver and gall tumors (16, including 14 liver carcinoma cells, 1 gallbladder carcinoma GBC-SD, and 1 intrahepatic cholangiocarcinoma cell RBE), hematopoietic neoplasms (43, including 12 lymphomas, 23 leukemia cell lines and 8 myelomas), brain tumors (27, including 14 gliomas, 3 cerebromas, 6 neuroblastmoas and 4 medelloblastomas), melanomas (10), motor system tumors (10, including 8 osteosarcomas and 2 rhabdomyosarcomas), renal cell carcinomas (21), bladder carcinomas (5), prostate carcinomas (12), head and neck tumors (17, including 6 oral carcinoma cell lines, 9 nasopharyngeal carcinomas and 2 thyroid tumor cell lines) and other tumors (11, including 2 adrenocortical carcinomas, 2 embryonal carcinomas, retinoblastoma cell line Y79, cecum adenocarcinoma HCE8693, duodenal carcinoma HuTu-80, skin squamous cell carcinoma A431, foreskin carcinoma HFF, fibrosarcoma HT1080 and adenocarcinoma cell F56). Numbers of authenticated cell lines and misidentified cell lines, including intra-species cross-contaminated and inter-species cross-contaminated cell lines in each category were showed in Fig. 2b.

### Cases of authenticated human tumor cell lines without public data

Among the 383 authenticated human cells, the STR profiles of 340 cell lines were consistent with those published in the DSMZ STR database. However, there were no published data for the remaining 43 cell lines that could be used for comparison (see Supplementary Table S2). Additionally, 18 of the 43 cell lines were newly established by CICR, and the corresponding primary tissues were preserved while STR profiling was performed. Therefore, despite the lack of published data, these 18 cell lines were verified to be authentic by comparison with primary tissues. 17 cell lines without primary materials for reference, including cells established in China and cells introduced from abroad. However, the STR profiles of these 17 cells were unique when compared with data published on the DSMZ STR database. The left 8 cells were derivatives of cell lines without public STR data, but their STRs were exact match (between 90 and 100% match) with that of parental lines. As a result, these 25 cells were provisionally considered to be correct.

### Cases of intra-species cross-contaminated human tumor cell lines

**Figure 3:**
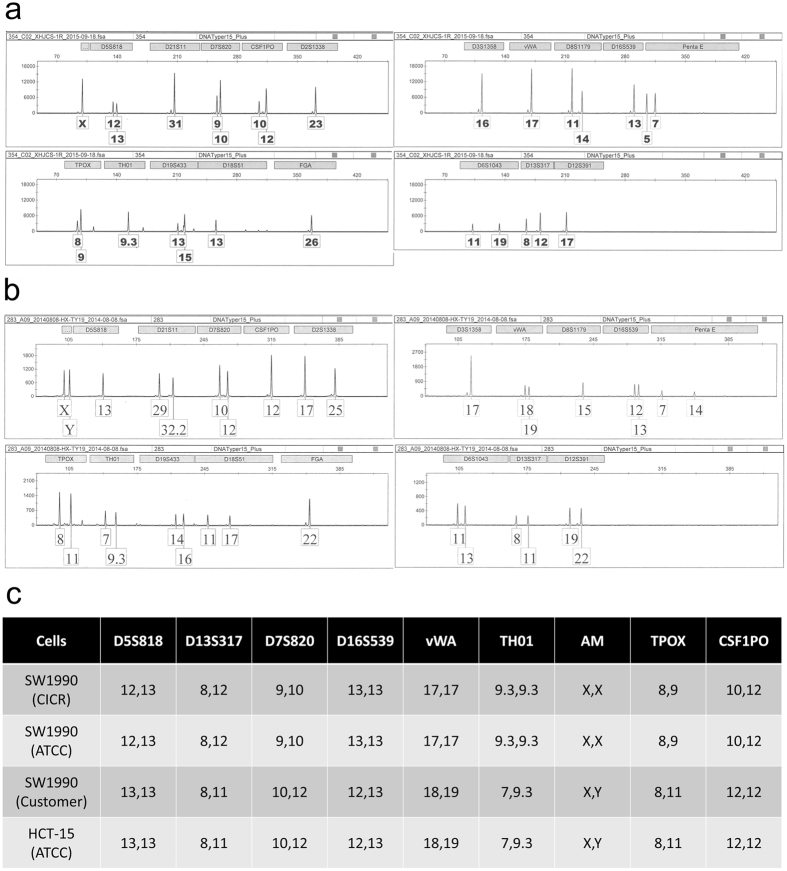
Electropherogram of SW1990 (human pancreatic carcinoma cell line) from two laboratories. (**a**) STR profile of SW1990 from China Infrastructure of Cell Line Resource (CICR), which is an authentic cell line. (**b**) STR profile of SW1990 from a customer’s laboratory, which is cross-contaminated by HCT-8 (human colorectal carcinoma cell line). (**c**) Comparison of STR profiles with ATCC.

Although they had the correct species (human) of origin, 70 (14.5%) cases were found to have the wrong STR profile, indicating that they were misidentified and had been replaced by another cell line. Detailed data are shown in Supplementary Table S3. Among these 70 misidentified human cell lines, 41 cell lines were used worldwide and 29 cell lines were established by Chinese scholars. 15 cell lines were in the Database of Cross-Contaminated or Misidentified Cell Lines of ICLAC (International Cell Line Authentication Committee), including the notable HeLa-replaced cell lines (ACC-2, ACC-M, KB and HEP-2)^8–11^, the glioma cell line SF767, which was substituted with the human cervical cancer cell line ME-180, and the nasopharyngeal carcinoma tumor lines CNE-1 and CNE-2, which are thought to be fusion cell lines between HeLa and another, unknown cell line^12, 13^. Another 14 misidentified cell lines were reported in some papers^5, 14, 15^. For example, the widely used glioma cell line U87MG is different from the original cells and it is likely to be a bona fide human glioblastoma cell line of unknown origin^14^. There remain 41 famous cell lines that are misused in China and lack reports of cross-contamination, including cells substituted by cells of the same tumor type and cells replaced by cells of different tumor types. In the former case it contains the human pancreatic cell line Capan-1 (identified as AsPC-1), esophageal carcinoma cell line KYSE410 (identified as KEYSE150), glioma cell line U251 (identified as GOS-3), lung cancer cell lines NCI-H3122 (identified as NCI-H1299) and so on. More cell lines belong to the latter case, such as the human pancreatic cancer SW1990 (identified as HCT-8, a colorectal carcinoma cell) (Fig. 3), the human retinoblastoma cell Y79 (identified as Raji, a human Burkitt’s Lymphoma cell line), QGP-1 which was established from a human pancreatic islet cell carcinoma and useful useful for studying the regulation mechanism of somatostatin secretion (identified as BxPC-3, a human pancreatic carcinoma cell line)^16^, the human pancreatic cancer cell BON-1 (identified as AGS, a gastric carcinoma cell), the human small cell lung cancer NCI-H446 and glioma cell line S4 (both identified as Hela, a cervical carcinoma cell line). Among the cells established by Chinese scholars, more than 65.5% (19/29) were replaced by HeLa cells, concluding human liver cancer cell lines BEL7402, QGY7701, QGY7703 and SMMC7721, esophageal squamous Cell Carcinoma cell EC9706, gastric cancer cells BGC823, MGC803 and SGC7901, lung cancer cells SPC-A1 and GLC-82, oral squamous cell carcinoma Tca-8113, ovarian carcinoma cells Ho8910 and Ho8910PM, nasopharyngeal carcinoma tumor lines CNE-1, CNE-2Z and 4 derivatives. However, MGC803 obtained from another laboratory showed different STR profile with Hela and other cells in DSMZ database, so this cell is considered as correct MGC803. Additionally, 2 (THP-21 and K562-21) of the 29 cross-contaminated cell lines may be caused by mislabeling. There was also cross-contamination among cell lines from the same depositor. Five lung cancer cell lines (lung adenocarcinomas LTEP-a1, LTEP-a2 and LTEP-a3, squamous cell carcinoma LTEP-S and small cell lung cancer LTEP-sm)^17, 18^ were reportedly sampled from 5 different patients; however, only 2 STR profiles were detected. Five human uveal melanoma cell lines (C918, M619, Mum-2B, Mum-2C and OCM-1A) were supplied by a single depositor. However, as reported, OCM-1 cells were originally cultured from a human choroidal melanoma specimen in 1985^19^, C918 and M619 cells were derived from patient tumors at the University of Iowa in the 1990s^20^ and Mum-2B and Mum-2C were derived from a heterogeneous tissue explant MUM-2^21^. These 5 cell lines should thus show 4 different STR DNA types, but only 2 STR profiles were detected. These cell lines are no longer suitable for research.

### Cases of inter-species cross-contaminated human tumor cell lines

**Figure 4:**
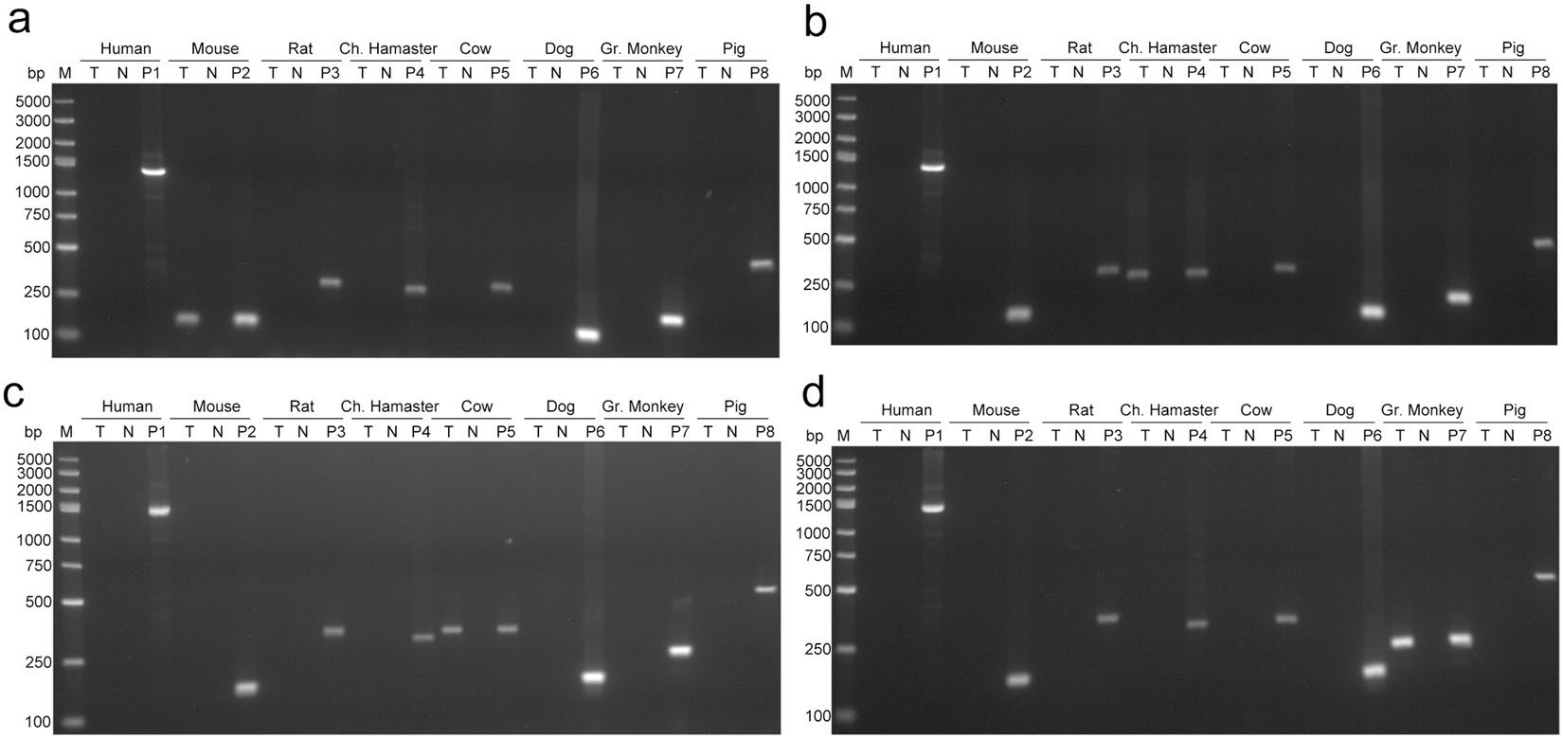
Cases of human cells fully substituted by animal cells. (**a**–**d**), by PCR-based species identification, Ramos (human Burkitt’s lymphoma B cells), A549-CP-H2 (human lung cancer cells with Cas9 stable expression), A427 (human lung cancer cells) and SCaBER (human bladder cancer cells) were cross-contaminated by cells of mouse, Chinese hamster, bovine and monkey origin, respectively. T, test sample; N, deionized water used as negative control; P, cell lines of corresponding species used as positive control separately, P1, RD (human rhabdomyosarcoma cell line); P2, Hepa 1–6 (mouse hepatocarcinoma cell line); P3, PC-12 (rat phaeochromocytoma cell line); P4, CHO (Chinese Hamster ovary cells); P5, MDBK (bovine kidney cell line); P6, MDCK (dog kidney cell line); P7, VERO (African Green Monkey kidney cell line); P8, LLC-PK1(pig kidney cell line); M, DNA marker; bp, base pairs.

When species identification was performed by PCR, 21 human cell lines were found to be fully substituted by another species such as rat, monkey, Chinese hamster, Syrian hamster or bovine (see Supplementary Table S4, Fig. 4). Some of these cell lines were further examined by STR profiling, and no signal was detected, as anticipated. Among these inter-species cross-contaminated human cell lines, 57.1% (12/21) were contaminated by mouse cells.

### Cases of cross-contaminating human tumor cell lines

**Figure 5:**
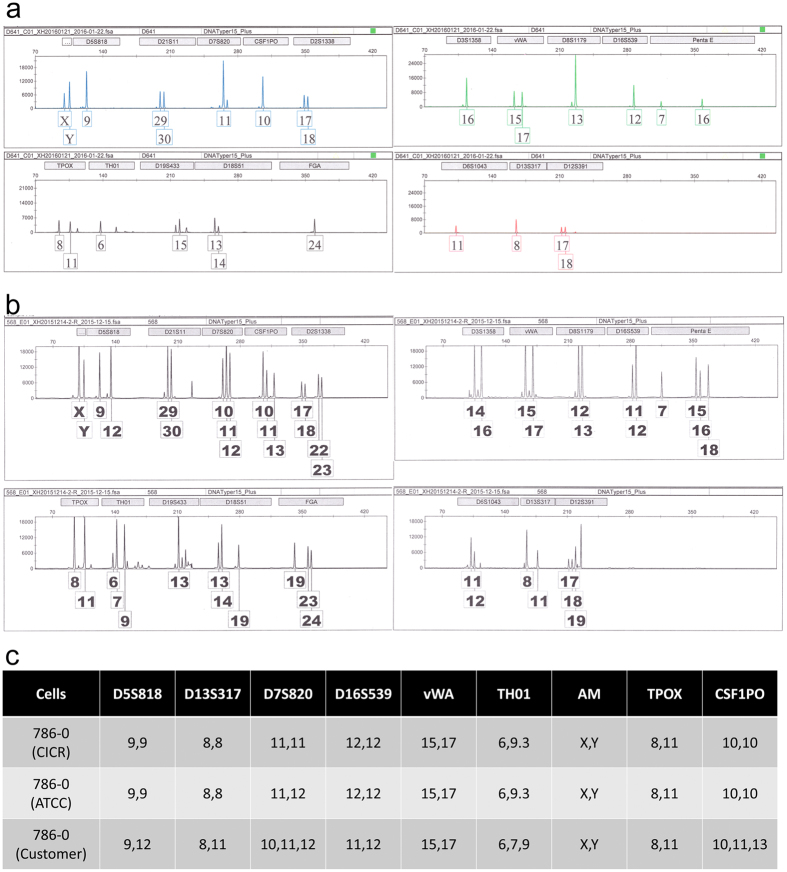
Electropherogram of 786–0 (human renal cell carcinoma) from two laboratories. (**a**) STR profile of 786–0 from China Infrastructure of Cell Line Resource (CICR), which is an authentic cell line. (**b**) STR profile of 786–0 from a customer’s laboratory, which is a mixture of multiple cells. (**c**) Comparison of STR profiles with ATCC.

There were 8 cell lines (see Supplementary Table S5) that were mixtures of multiple cell lines. When analyzed by STR profiling, 3 cell lines (803-Luc2-TdT, 803-mCherry and 786–0) had 3 or 4 types of copy numbers in more than 5 STR loci (Fig. 5). By comparison with the STR database or with STR data for these cell lines collected from earlier passages, it was found that these cell lines were cross-contaminated by other cells in culture.

**Figure 6:**
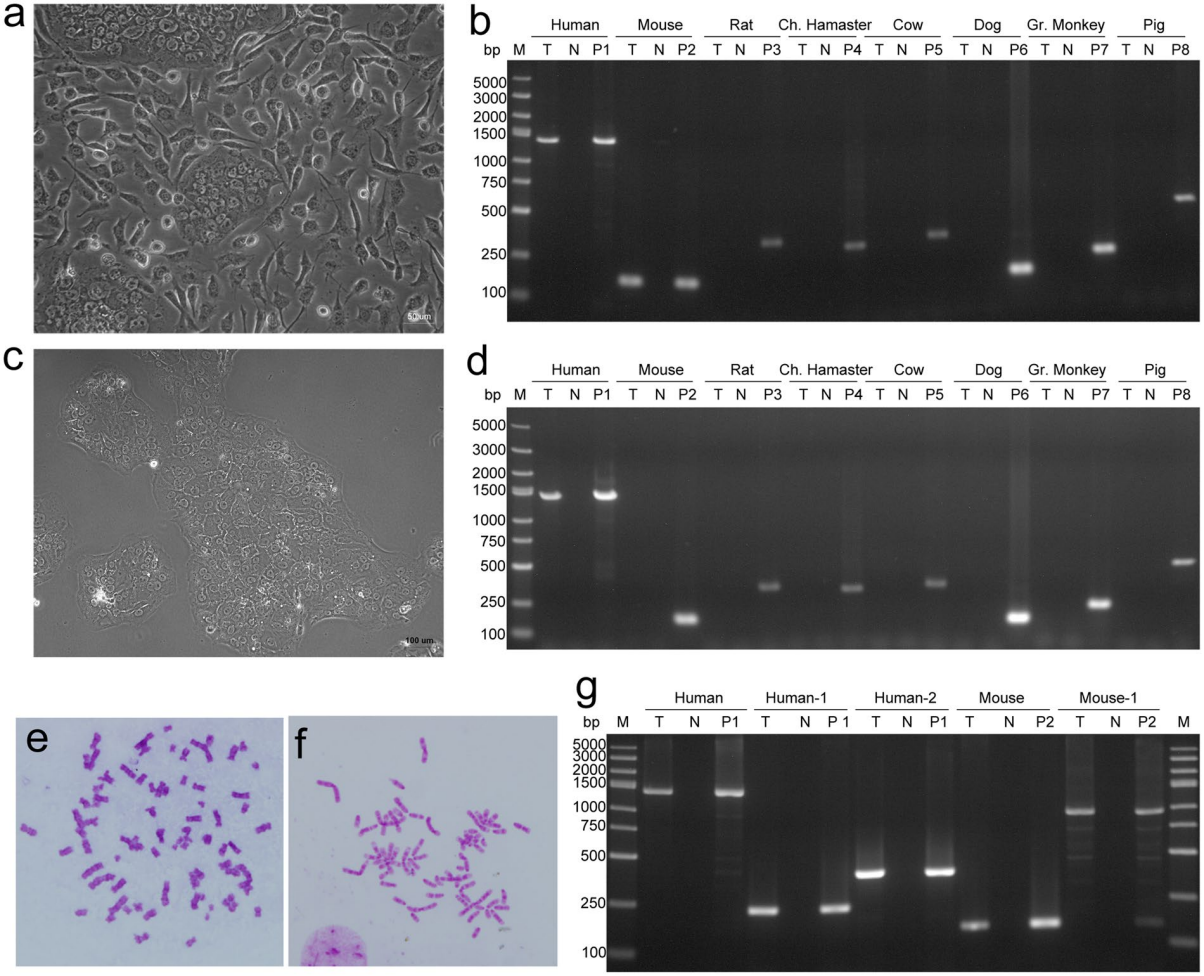
Species identification of BeWo (Human choriocarcinoma cells). (**a**) Two distinct morphology was found in the microscopic view of BeWo(A) from A laboratory; (**b**) human and mouse specific bands were both found by PCR-based species identification in BeWo(A); (**c**) only one morphology was found in the microscopic view of BeWo(B) from B laboratory; (**d**) BeWo(B) was identified as human cells by PCR-based assay; (**e**) human chromosome was found in the metaphase spreads of BeWo(A); (**f**) mouse chromosome was found in the metaphase spreads of BeWo(A); (**g**) The result of PCR with species-specific primers targeting different DNA loci confirmed that BeWo(A) was a mixture of human and mouse cells. T, test sample; N, deionized water used as negative control; P, cell lines of corresponding species used as positive control separately, P1, RD (human rhabdomyosarcoma cell line); P2, Hepa 1–6 (mouse hepatocarcinoma cell line); P3, PC-12 (rat phaeochromocytoma cell line); P4, CHO (Chinese Hamster ovary cells); P5, MDBK (bovine kidney cell line); P6, MDCK (dog kidney cell line); P7, VERO (African Green Monkey kidney cell line); P8, LLC-PK1(pig kidney cell line); M, DNA marker; bp, base pairs.

**Figure 7:**
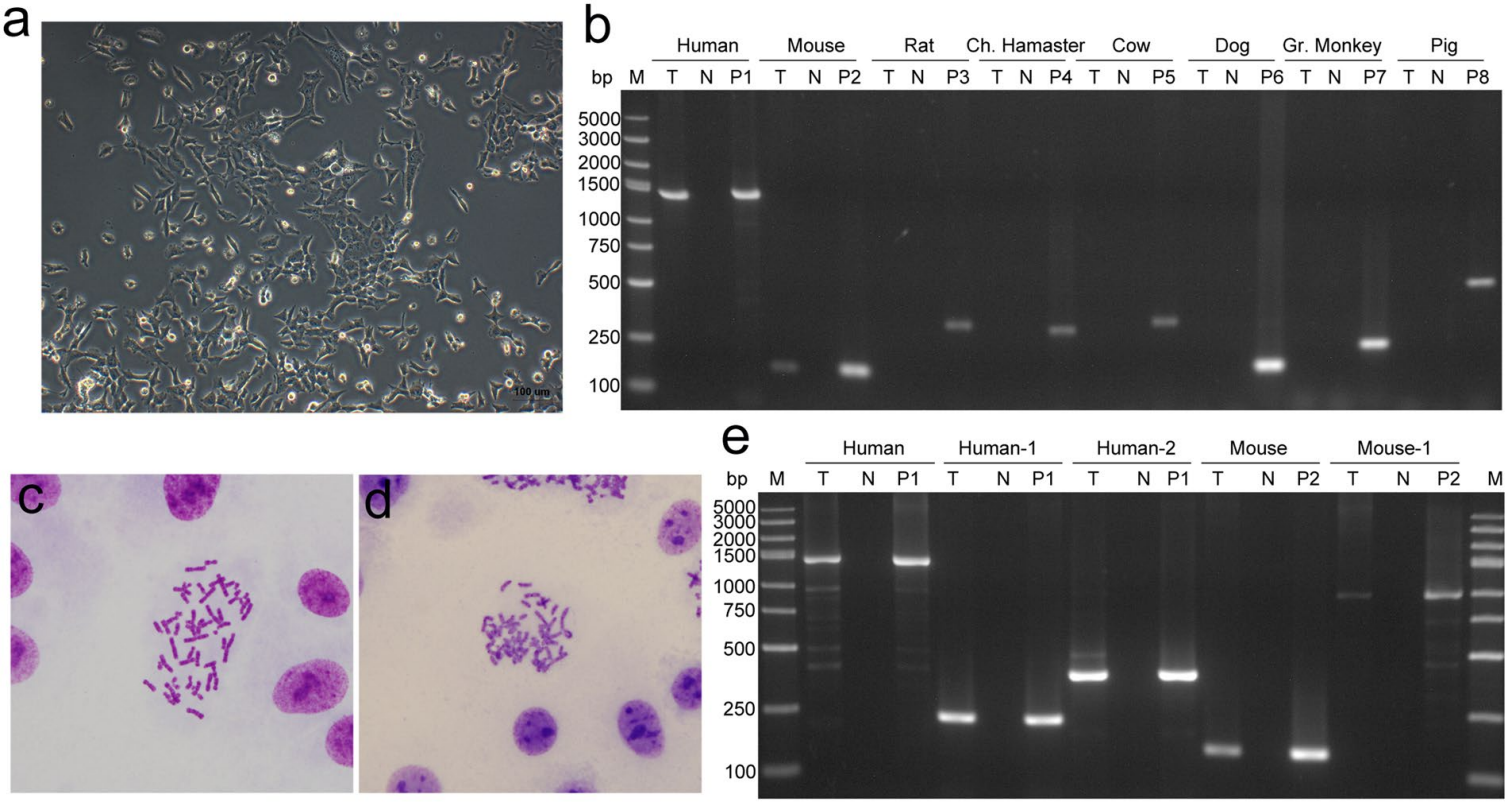
Species identification of PG-LH7 (Human lowly metastatic lung cancer cells). (**a**) No distinct morphological variation was found in the microscopic view of PG-LH7; (**b**) human and mouse specific bands were both found by PCR-based species identification in PG-LH7. (**c**) Human chromosome was found in the metaphase spreads of PG-LH7; (**d**) mouse chromosome was found in the metaphase spreads of PG-LH7; (**e**) The result of PCR with species-specific primers targeting different DNA loci confirmed that PG-LH7 was a mixture of human and mouse cells. T, test sample; N, deionized water used as negative control; P, cell lines of corresponding species used as positive control separately, P1, RD (human rhabdomyosarcoma cell line); P2, Hepa 1–6 (mouse hepatocarcinoma cell line); P3, PC-12 (rat phaeochromocytoma cell line); P4, CHO (Chinese Hamster ovary cells); P5, MDBK (bovine kidney cell line); P6, MDCK (dog kidney cell line); P7, VERO (African Green Monkey kidney cell line); P8, LLC-PK1(pig kidney cell line); M, DNA marker; bp, base pairs.

Notably, the human cell lines that were contaminated, but not fully substituted, by cells from other species showed correct STR profiles. In this study, BeWo, a human trophoblastic choriocarcinoma cell line^22^ shown in Supplementary Table S5, was collected from a well-established lab, and it had 2 distinct morphologies (named as BeWo(A)). Figure 6a shows a microscopic view upon arrival. The 2 constituent morphologies were carefully maintained, although one detached much more quickly than the other. When evaluated by STR, the profile of this BeWo sample was consistent with that of BeWo in ATCC and BeWo from another laboratory (named as BeWo(B)), which showed only one morphology under a microscope (Fig. 6c). However, when the cell line species was confirmed by PCR, BeWo(A) was found to be a mixture of human and mouse cells and BeWo(B) was verified as a human cell line (Fig. 6b,d). This result was confirmed by chromosome analysis and PCR method with human- and mouse-specific primers targeting other DNA loci (Fig. 6e,f). Some cell lines, such as the human lowly metastatic lung cancer cell line PG-LH7, lacked 2 distinct cell morphologiesand showed correct STR profiles, but they were still mixed with cells from other species (Fig. 7).

## Discussion

Tumor cell lines are often fundamental materials in life science and basic medical research, and the use of unverified cell lines may result in invalid data, leading to unreliable results^23^. Cross-contamination, in which the contaminant is another cell line, was first recognized in the 1950s but still remains a serious issue today^1^. Many studies have shown that a surprisingly large number of cell lines have been contaminated; they are often contaminated by older, more well-established cell lines^1, 3, 24–28^. For example, ECV304, a cell line from the human umbilical vein, had been established and maintained for more than 5 years (180 generations and 900 population doublings) when reported in 1990 by Japanese scholars. However, in 1999, Dirks *et al*. reported that ECV304 was substituted with T24 human bladder carcinoma cells^29–31^. From 2004 to the present, more than 400 articles have used ECV304 as an endothelial cell; nearly 70% of these were published by Chinese scholars. In 2009, *Nature* announced: “It is time for all involved to tackle the chronic scandal of cell line contamination”. Today, most notable publications require that all cells lines used in a paper are verified before use, but this QA step has not been required in China. The government began taking measures to standardize research materials and resources more than 15 years ago by establishing the National Science and Technology Infrastructure (NSTI). As part of the NSTI, the China Infrastructure of Cell Line Resource (CICR) has focused on the integration, standardization and sharing of cell lines. Of all quality control measures in the system, the quality analysis of cell lines is our priority. In this paper, we report the authentication of cell lines. Among 482 human tumor cell lines tested in our study, there were 20.5% (99/482) of misidentified cell lines, which is lower incidence than that reported by Ye *et al*. (25.0%, 95/380)^5^ and Huang *et al*. (46.0%, 128/278)^15^ because of different statistical range and calculating method, as mentioned in “Samples and genomic DNA extraction” in the Methods. Some were misidentified when introduced to China long ago, such as the famous HeLa contaminants KB and Hep-2, while some cells may have been cross-contaminated during culture in China. For example, Molt-4 (human leukemia cell line) and SK-OV-3 (human ovarian carcinoma cell line) have been accepted as authentic^32^ and their STR profiles are included in the DSMZ database. Meanwhile, authenticated Molt-4 and SK-OV-3 can be found in our center or other laboratories. So the problem is likely to be confined to the lab that supplied the sample to us for testing, or more broadly to Chinese labs who are sharing a particular stock that is misidentified. Some cell lines established by Chinese scholars were cross-contaminated at the beginning of culture, such as BCA4, which STR profile is different from that of donor tissue. For researchers in China, it is of the utmost importance to ensure that the cell lines in use have a well-defined origin and are routinely re-analyzed to identify possible areas of contamination. Researchers can find well-authenticated cell lines from the China Infrastructure of Cell Resource (CICR).

The risk of contamination by unrelated cells is a potential and often recurrent problem. In this study, we detected more than one case of cells from one depositor that were cross-contaminated with each other. Cross-contamination may arise due to several causes, including use of unchanged tips, sharing media and reagents among cell lines and use of mitotically inactivated feeder layers or conditioned medium, and mislabeling^1^. Good lab practices (GLP) for tissue culture, including strict aseptic technique and vigilant observation of cellular morphology, are essential for preventing cross-contamination. When one cell line (termed A) is contaminated by another cell line (termed B), if B cells grow more quickly, A will be replaced by B in a few generations. If A and B have similar growth rates, GLP can help maintain the original A cells, and single-cell cloning can ensure the preservation of the A cell line. In the same way, if A cells are contaminated by B cells that are sensitive to trypsin digestion and easy to detach from the plate, A will be substituted by B cells. In addition, differences in morphology can arise from multiple clones in the original mass culture that evolve with passaging^33^, and can occur by culture conditions over time that could be related to causing differentiation of cancer stem cells within the population. These different populations may be more or less evident depending on the core stock used, number of passages, and the culture conditions prior to each vial preservation. Furthermore, heterogeneity occurs when cells are cultured over extended periods of time, subjected to differing culture conditions or are genetically unstable^32^. Therefore, to avoid phenotypic or genotypic drifting, it is better to adhere to the original way of nurturing the cells. With careful daily morphological examination, if two distinct morphologies are observed, the cell identity must be re-authenticated to eliminate cross-contamination. Notably, loss of heterozygosity, microsatellite instability, aneuploidy in tumor cell lines and cross-contamination make authentication problematic. It is reported that a cutoff of 70% identity for 16-locus STRs (85% for 8-loci) is needed to confirm cell line identity^32^.

Cell cross-contamination includes inter-species and intra-species contamination. Today, isoenzyme electrophoresis, chromosome analysis, DNA barcoding and PCR-based assays are used for detecting inter-species contamination^1, 9, 34–40^. In our center, species-specific primers were used to easily distinguish among human, mouse, rat, Chinese hamster, Syrian hamster, bovine, dog, monkey, pig and rabbit DNA using agarose gel electrophoresis. Some of the primers targeted at the conserved sequences of the cytochrome C oxidase I gene (COI), described as “barcode region” for animal species^34, 36^. A PCR-based assay is a simple, quick, accurate and cost-effective way to authenticate the species origin of a cell line and evaluate for inter-species cell line contamination in a single test. To confirm or distinguish a more broad range of animal species, chromosome analysis or sequencing of “DNA barcode” regions can be employed^1, 34^. In this study we found that cross-contamination of human and murine cells was the most common type of inter-species contamination because human and murine cell lines are the most commonly used types of cell lines. In recent years, STR profiling has been suggested as a method for authenticating human cell lines. By STR typing, it is easy to identify human cells that have intra-species contamination or that have been fully replaced by animal cells. However, human cells that were partially contaminated by animal cells always showed correct STR profiles. Therefore, we recommend first performing species verification and then STR profiling for human cell identification.

The HeLa cell line was the first human cell line; it was established in 1951 from a cervical tumor biopsy taken from Henrietta Lacks, a working-class African-American woman living near Baltimore^9, 26, 41, 42^. It was once the most widely used cell line due to its hardiness and rapid growth rate. However, it has been demonstrated that HeLa cells have contaminated many other cell lines; they have overgrown and completely replaced such original cell lines as WISH, KB, Hep-2, Chang Liver, INT407 and the adenoid cystic carcinoma cell lines ACC-2, ACC-3 and ACC-M^5, 8–11^. From 1969 to 2004, 220 publications in the PubMed database were found to have used HeLa-contaminated cell lines^42^. In this study, we found 25 HeLa-contaminated tumor cell lines, 19 of which were established by Chinese scholars and include the gastric cancer cell lines BGC823, MGC803 and SGC791; the liver cancer cell lines BEL7402, QGY7701, QGY7703 and SMMC7721; and the ovarian cancer cell lines Ho8910 and Ho8910PM, which are widely used in anti-cancer research in the corresponding cancer fields. In addition, HCT-8, HCT116 and NCI-H157 contaminants were also common in cases of human intra-species cross-contamination.

In conclusion, species identification followed by STR profiling is a valuable way to authenticate cell identity. Among 482 tested tumor cell lines used in China, we detected 99 misidentified cell lines, corresponding to an incidence of 20.5%. We recommend that scholars employ only authenticated cell lines for research and perform GLP during the process. We also recommend that scholars re-examine previously stored laboratory stocks of cell lines, especially the contaminated cell lines listed here. In Chinese cell resource centers, more vigorous efforts should be taken to propagate the importance and benefits of cell line authentication.

## Methods

### Samples and genomic DNA extraction

Cell pellets from 482 human tumor cell lines were collected from 2008 to 2016 by the Cell Resource Center of Peking Union Medical College (PUMC) or from customers’ laboratories. Cells with the same name and same STR profiles are considered as one cell line, although they were obtained from different laboratories, and cells with the same name but distinct testing results (STR profiles or species) were considered as different cells. These cell lines were established from patients with a variety of tumors, including lung carcinoma, breast carcinoma, colorectal carcinoma, liver carcinoma, leukemia, melanoma, glioma, and so on. Total genomic DNA was extracted using the PureLink® Genomic DNA Mini Kit (K1820-02, Invitrogen), and DNA yields were assessed using a NanoDrop 2000 (Thermo, USA). DNAs were first tested cell species by PCR method and then examined by STR profiling.

### Cell species identification by PCR

Cell species identification by PCR was performed according to a previously reported method^43^. In brief, genomic DNA samples were separately amplified using multiple species-specific primers(Supplementary Table S6 and S7), and specific bands were examined by agarose gel electrophoresis. The PCR reaction mixture (20 µl) contained 50 ng of genomic DNA from the test sample, 10 µl of 2X EasyTaq Mix (AS111, Transgen), 1 µl of primers (5 µM each each) and deionized water. Deionized water was used as template of negative control, and the genomic DNA of RD (human rhabdomyosarcoma cell line), Hepa 1–6 (mouse hepatocarcinoma cell line), PC-12 (rat phaeochromocytoma cell line), CHO (Chinese Hamster ovary cells), MDBK (bovine kidney cell line), MDCK (dog kidney cell line), VERO (African Green Monkey kidney cell line), LLC-PK1(pig kidney cell line), BHK-21 (Syrian Hamster kidney cells) and CCC-SMC-1 (rabbit aortic smooth muscle cells) were used as templates of positive control for each species separately. The PCR program was as follows: 95 °C for 5 min; 26 cycles of 95 °C for 30 s, 56 °C for 30 s and 72 °C for 1 min; and 72 °C for 10 min. The PCR products were examined by 1.2% agarose gel electrophoresis.

### Chromosome analysis

Cells in an exponential growth phase were karyotyped using a standard air-dried method after treatment with 0.01 μg/ml colcemid for 2 hours. Metaphase spreads were observed and photographed under oil microscope.

### STR profiling

STR loci and the amelogenin sex-determining marker were amplified using the AmpFlSTR™ Identifiler® Plus PCR Amplification Kit (Applied Biosystems, USA) (detecting amelogenin, D5S818, D21S11, D7S820, CSF1PO, D2S1338, D3S1358, vWA, D8S1179, D16S539, TPOX, TH01, D19S433, D18S51, FGA and D13S317) or the DNA Typer^TM^15 plus direct kit (Institute of Forensic Science, Ministry of Public Security, China)(detecting the loci listed above plus Penta E, D6S1043 and D12S391) according to the manufacturer’s instructions in a GeneAmp®PCR system 9700. Electrophoretic analysis was performed using a 3730/3130xl DNA Analyzer (Applied Biosystems, USA). After electrophoresis, the data were analyzed with Gene Mapper® ID-X Software v1.2 (Applied Biosystems, USA) to categorize peaks by size in relation to an internal standard allelic ladder.

### Data analysis

STR data were analyzed using the DSMZ (German Collection of Microorganisms and Cell Cultures) online STR database (http://www.dsmz.de/fp/cgi-bin/str.html), which includes STR data sets for 2,455 human cell lines from ATCC, DSMZ, JCRB and RIKEN. After registration, our STR data were entered for matching with the authenticated cell lines listed in the database^44^. At the same time, STR profiles of cell lines were compared to additional reference where available, such as that of donor tissue. Furthermore, all cell lines were checked against database of cross-contaminated or misidentified cell lines on ICLAC (International Cell Line Authentication Committee) (Version 8.0, http://iclac.org/databases/cross-contaminations/).

### Data availability

All data generated or analysed during this study are included in this published article (and its Supplementary Information files).

## Electronic supplementary material

Supplementary information

Supplementary table S1-S5
