# Supplementary material for: A Combination of Species Identification and STR Profiling Identifies Cross-contaminated Cells from 482 Human Tumor Cell Lines
Source: Sci Rep. 2017 Aug 29;7:9774. doi: 10.1038/s41598-017-09660-w (PMC5575032; doi:10.1038/s41598-017-09660-w)
Supplement: Supplementary file 1 — Supplementary information [file 41598_2017_9660_MOESM1_ESM.pdf]

Supplementary Information

Title: A Combination of Species Identification and STR Profiling Identifies Cross-contaminated Cells from 482 Human Tumor Cell Lines

Authors: Xiaocui Bian, Zhenli Yang, Hailiang Feng, Hao Sun[1] & Yuqin Liu

Supplementary Table S1. List of authenticated human tumor cell lines (.xls) .
Supplementary Table S2. List of authenticated human tumor cell lines without public data(.xls) .
Supplementary Table S3. List of intra-species cross-contaminated human tumor cell lines(.xls) .
Supplementary Table S4. List of inter-species cross-contaminated human tumor cell lines(.xls) .
Supplementary Table S5. List of cross-contaminating human tumor cell lines(.xls) .

Primer Names	Sequence (5'-3')	Gene	Amplicon Size(bp)	Reference
Human-F	CAAGACAGGTTTAAGGAGACCA	c-globin	1411	PMID: 14640788
Human-R	GCAGAATCCAGATGCTCAAGG			
Mouse-F	ATTACAGCCGTACTGCTCCTAT	cox Ⅰ	150	PMID: 11848419
Mouse-R	CCCAAAGAATCAGAACAGATGC			
Rat-F	AGACACTCTGACGACTGTCAACA	D4Rhw5	317	UniSTS: 463382
Rat-R	CATGGTAGAGAAAATCTGTTCCG			
Chinese Hamster-F	GTGACCCATATCTGCCGAGAT	Cytochrome b	293	PMID: 11848419
Chinese Hamster-R	CATTCTACTAGGGTGGTGCCC			
Syrian Hamster-F	AGGTGATCCACTCCTTCGCT	c-globin	~1500	PMID: 14640788
Syrian Hamster-R	TGTTCTCTAGGGAACAAGTGACTT			
Monkey-F	CCTCTTTCCTGCTGCTAATG	cox Ⅰ	222	PMID: 11848419
Monkey-R	TTTGATACTGGGATATGGCG			
Rabbit-F	CGGGAACTGGCTTGTCCCCCTG	cox Ⅰ	151	PMID: 11848419
Rabbit-R	AACAGTTCAGCCAGTCCCCGCC			
Pig-F	ACTGCCAGCAGCCTAAATGTAT	MARC_67887-67888:1205421730:3	517	UniSTS: 516113
Pig-R	TCCCTAACTTGCCAGTCTTAGC			
Bovine-F	TCACTGGCTTACAACTAGGG	BT225	272	UniSTS: 64730
Bovine-R	TGGAGATGAGTTTGACTAAG			
Dog-F	GAACTAGGTCAGCCCGGTACTT	cox Ⅰ	153	PMID: 11848419
Dog-R	CGGAGCACCAATTATTAACGGC			

Supplementary Table S6. Sequences of Species-Specific Primers for Routine Cell Species Identification.

Primer Names	Sequence (5’-3’)	Gene	Amplicon Size(bp)	Reference
Human-1-F	TTCGGCGCATGAGCTGGAGTCC	cox Ⅰ	228	PMID: 11848419
Human-1-R	TATGCGGGGAAACGCCATATCG			
Human-2-F	ATTACAGCCGTACTGCTCCTAT	cox Ⅰ	391	PMID: 17934781
Human-2-R	CCCAAAGAATCAGAACAGATGC			
Mouse-1-F	AGACACTCTGACGACTGTCAACA	c-globin	999	PMID: 14640788
Mouse-1-R	CATGGTAGAGAAAATCTGTTCCG			

Supplementary Table S7. Sequences of Human- and Mouse-Specific Primers for Identification of Suspicious Cross-contamination of human and mouse cells.

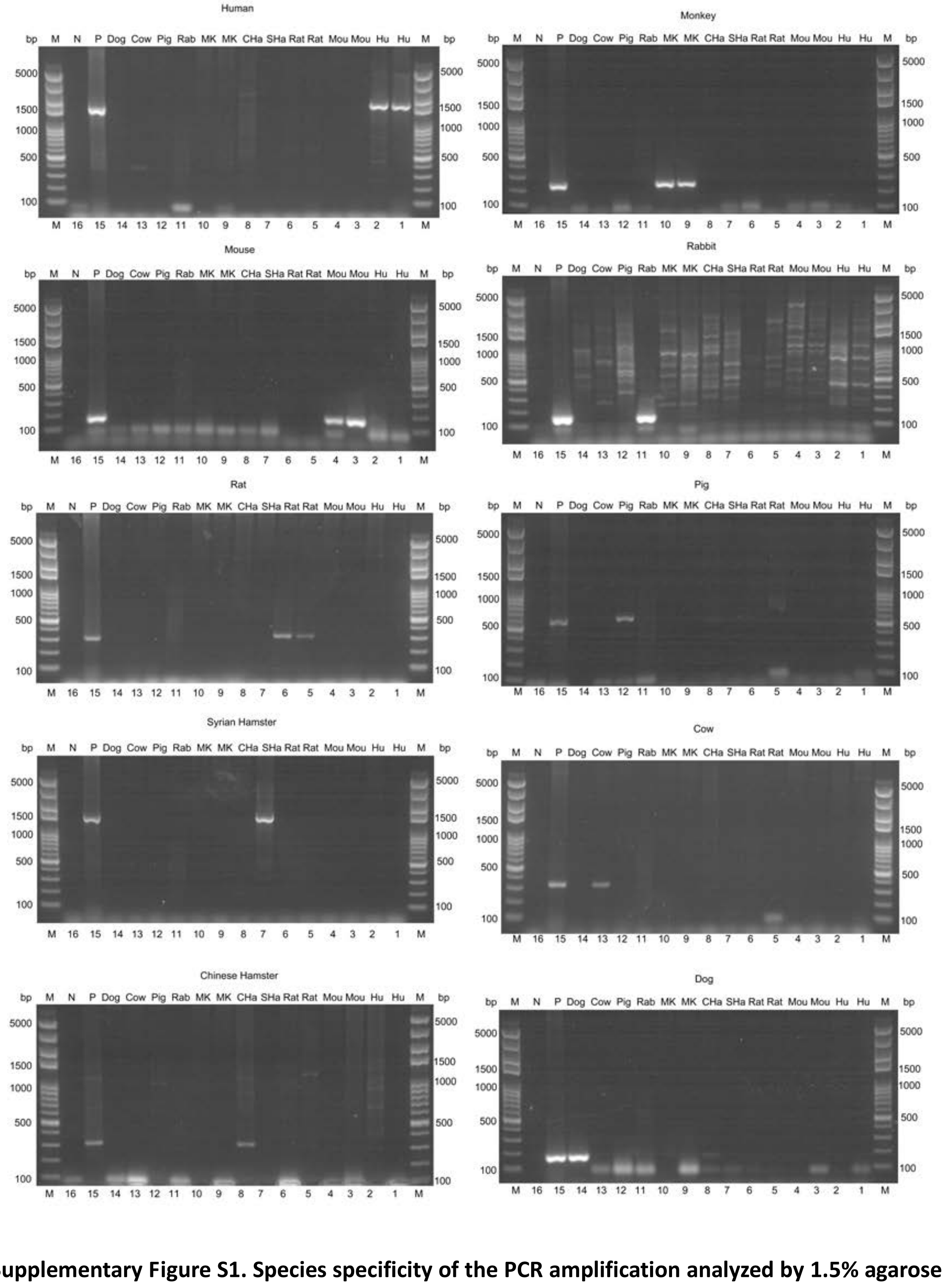

Supplementary Figure S1. Species specificity of the PCR amplification analyzed by 1.5% agarose gel electrophoresis. Primer pairs of 10 species were tested: human, mouse, rat, Syrian hamster, Chinese hamster, African green monkey, rabbit, pig, cow and dog. DNA extracted from 14 cell lines were used as templates of the PCR amplification. Meanwhile, the mixed DNA and water were used as positive control or negative control. On each gel, samples are loaded in the same order(from right to left): marker, products of PCR amplification with RD(human), SMMC 7721(human), Hepa 1-6(mouse), Neuro-2a(mouse), PC12(rat), RINm5F(rat), BHK-21(Syrian hamster), CHO(Chinese hamster), CV-1(African green monkey), VERO(African green monkey),

CCC-SMC-1(rabbit), LLC-PK1(pig), MDBK(bovine), MDCK(dog), positive control(mixed DNA) and negative control(water).

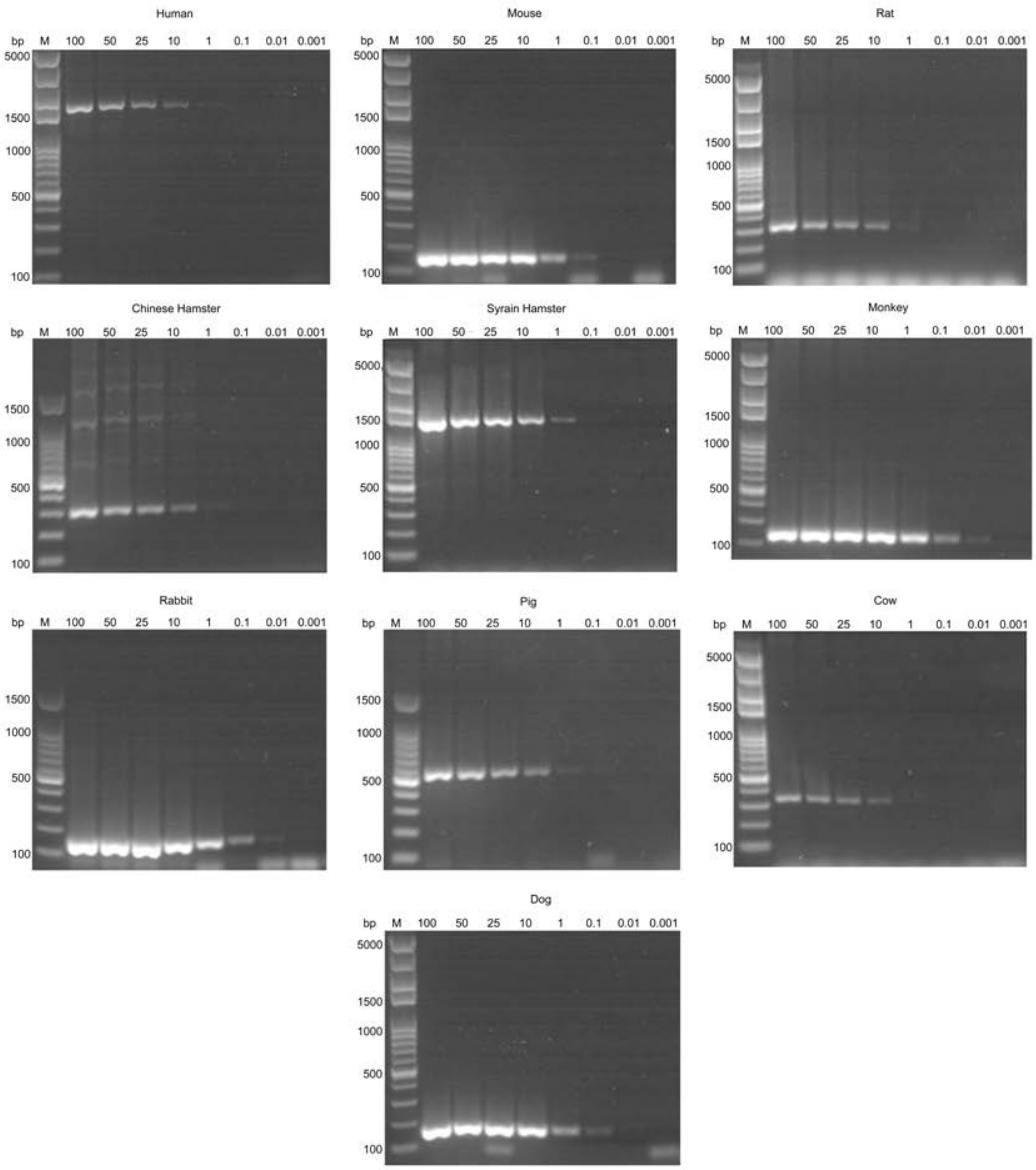

Supplementary Figure S2. Sensitivity of the PCR amplification analyzed by 1.5% agarose gel electrophoresis. 100ng、50ng、25ng、10ng、1ng、0.1ng、0.01ng and 0.001ng genomic DNA were amplified by PCR with corresponding primers, and then the amplified DNA fragments were run on a 1.5% agarose gel. DNA of each species was extracted from the following cell lines: RD(human), Hepa 1-6(mouse), PC12(rat), CHO(Chinese hamster), BHK-21(Syrian hamster), VERO(African green monkey), CCC-SMC-1(rabbit), LLC-PK1(pig), MDBK(Bovine) and MDCK(Dog).

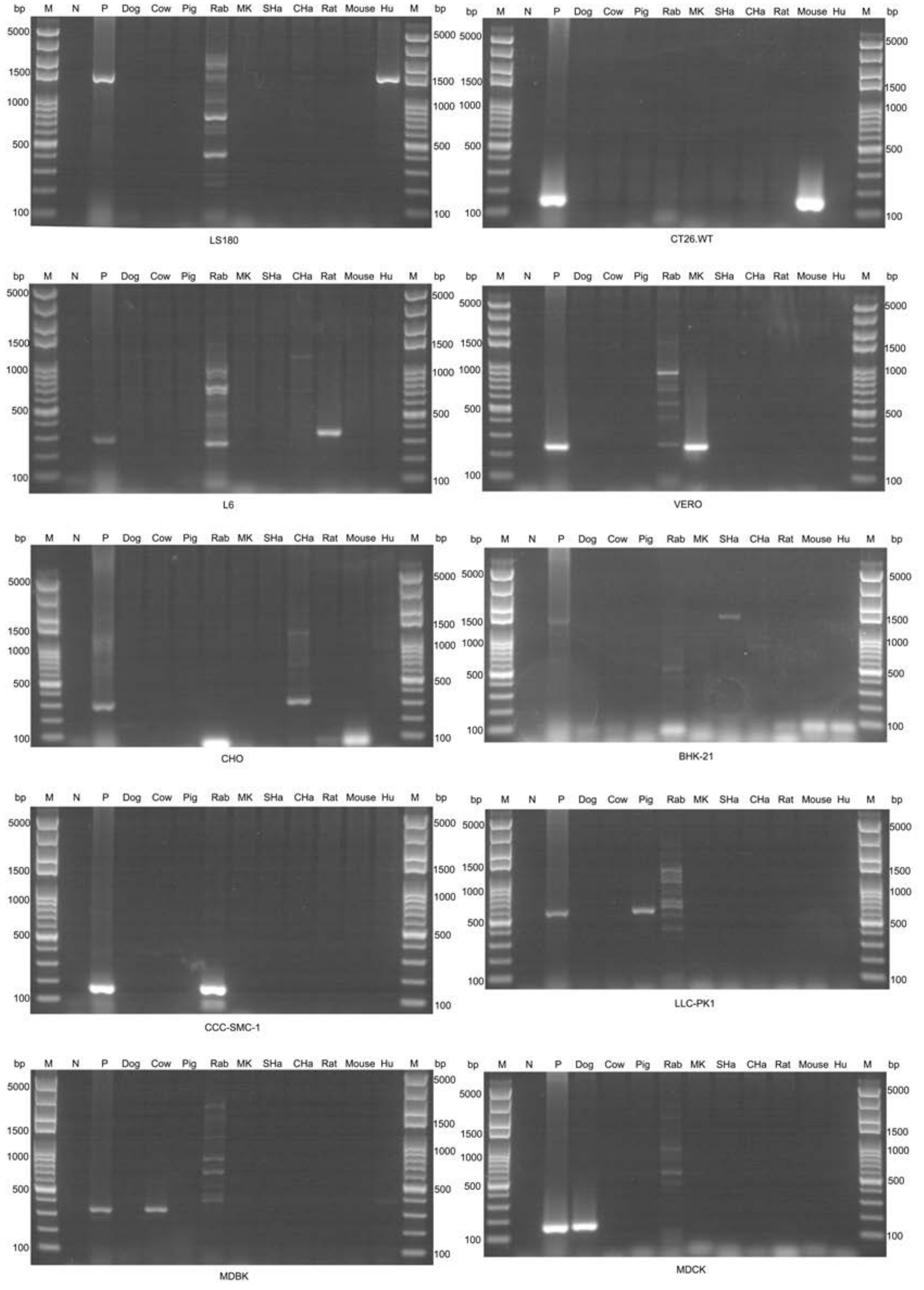

Supplementary Figure S3. Gel electrophoresis of the PCR products for 10 species. 50~100ng DNA extracted from ten cell lines was amplified with 10 species-specific primer pairs, and the amplified products were run on 1.5% gel. Ten cell lines showed here are LS180(human), CT26.WT(mouse), L6(rat), VERO(African green monkey), CHO(Chinese hamster), BHK-21(Syrian hamster), CCC-SMC-1(Rabbit), LLC-PK1(pig), MDBK(bovine) and MDCK(Dog). On each gel, the amplified products of 10 species are loaded in the same order(from right to left):human, mouse,

rat, Chinese hamster, Syrian hamster, African green monkey, rabbit, pig, bovine and dog. P, positive control(mixed DNA); N, negative control(water); M, marker; bp, base pairs.
